# Anesthetic Management of an Obstetric Patient with Behçet's Disease Complicated by Transverse Myelitis

**DOI:** 10.1155/2022/3082743

**Published:** 2022-02-24

**Authors:** Sara Alwatban, Etedal AlAamri, Abdullah Alraffa, Mona Alkhawajah

**Affiliations:** ^1^King Faisal Specialist Hospital and Research Centre (KFSH&RC), Makkah Al Mukarramah Branch Rd, Al Mathar Ash Shamali, Riyadh 11564, Saudi Arabia; ^2^Johns Hopkins Aramco Healthcare (JHAH), 6th Street, Gharb Al Dhahran, Dhahran 34465, Saudi Arabia

## Abstract

Transverse myelitis is an acute inflammation of the spinal cord. Its annual incidence is 1–8 per million. Behçet disease is also a rare autoimmune disease. Transverse myelitis can be a manifestation of neuro-Behçet's disease. For those two rare diseases to present in one patient sets a challenge in anesthetic management. Up to our knowledge, our approach to managing these cases has not been reported in the literature. We present the case of a 37-year-old female patient in her 34^th^ week of pregnancy, showing manifestations of neuro-Behçet's disease and recurrent transverse myelitis. She presented to the preanesthesia clinic on a wheelchair with worsening of baseline right lower limb weakness. The patient elected for neuroaxial anesthesia, and the procedure was conducted without complications. The patient was followed up for 6 months. The neuroaxial approach was successful. Patients with neuro-Behçet's disease and transverse myelitis can be safely managed with epidural anesthesia.

## 1. Introduction

Behçet's disease (BD) is a multisystem vasculitis of rare occurrence, and its clinical diagnosis is based on the signs and symptoms presented by the patient. The signs commonly include oral ulcers recurring ≥3 times per month, along with other systemic manifestations such as genital ulcers, dermal lesions, and ophthalmic lesions [1–3]. Neuro-Behçet's disease (NBD) is a complication of Behçet's disease [4]. Neurological symptoms are particularly relevant, considering that NBD sometimes involves the spinal cord [5]. Transverse myelitis (TM) is a rare inflammatory demyelinating condition that primarily affects the spinal cord; it is also a complication of BD [6]. Its annual incidence does not exceed 1–8 cases per million [7]. The disorder results in a spectrum of autonomous, motor, and sensory complications [6]. Despite the scarcity of clinical and empirical data, TM and BD have significant implications in clinical care and practice. Patients with BD or TM who require surgery may face complications during anesthesia [8].

Anesthesia approaches in managing patients with TM, BD, and NBD are rarely reported in the literature, specifically in a parturient. In this case report, we discuss a multidisciplinary approach for the management of such cases. To our knowledge, this is the first report on an obstetric patient with this presentation who was managed using epidural anesthesia. This manuscript adheres to the applicable CARE guidelines. The most optimal management of anesthesia in pregnant women with chronic or acute TM and BD is yet to be determined. A review of the literature reveals that general anesthesia in neuroaxial approaches is avoided in such patients; however, a clear explanation is not provided. Written informed consent was obtained from the patient for the publication of this case report. This research was approved by the Research Advisory Council and the Ethical Committee at King Faisal Specialist Hospital and Research Centre (PUB#2215037).

## 2. Case Report

This report describes a 37-year-old woman, Gravida 5 para 3 plus 1, at 34 weeks of gestation, with recently diagnosed BD, showing elevated B51 marker levels and recurrent painful genital ulcers associated with recurrent TM affecting T3–T7 levels, autoimmune thyroiditis with normal thyroid function, hypercholesterolemia, osteoporosis with ongoing vitamin D supplement use, and a history of smoking and mild bronchial asthma. The patient had no family history of BD or TM. Her first delivery was under general anesthesia, while her second and third were performed under spinal anesthesia.

The patient had a history of relapses in the form of dermatomal hyperesthesia lasting days to weeks. In October 2018, magnetic resonance imaging (MRI) revealed further enhancement of segments from T3 to T7. Soon after, the patient complained of recurrent stabbing pain in the back, resulting in worsening right lower limb weakness requiring a wheelchair and thereby, interference with daily activities. The patient experienced stabbing pain running down the course of her nerve injury. However, no numbness or orthostatic hypotension was noted.

During her visit to the preanesthesia clinic, she weighed 120 kg, was 161 cm tall, and had a body mass index of 45. On examination, she had a competent airway with no evidence of oral ulcers noted, her cardiovascular and respiratory functions were within normal limits, and there were no signs of autonomic dysfunction. During neurological examination, the patient was awake, alert, and oriented to time, place, and person, obeying simple commands and understanding complex ones; her speech was fluent and comprehensive. Motor nerve examination revealed a normal tone with full power in all extremities except her right hip flexor 2/5, right knee flexor 2/5, and right knee extension 4/5. However, since the patient still presented symptoms and signs of active TM and NBD, the anesthesia team decided to repeat the spinal MRI; however, no new findings were noted. After discussing the advantages and disadvantages of both general anesthesia and epidural anesthesia, a decision was made to use epidural anesthesia with an assisted bilevel positive airway pressure machine present on standby in the operating room for further respiratory support if needed.

In 2019, the patient was scheduled for an elective cesarean section. After ultrasound spine visualization, she was administered fentanyl (100 mg), morphine (3 mg), lidocaine 2% (11 mL), and bupivacaine 0.5% (4 mL) through an epidural catheter. She was repositioned after placing her in the supine position with right uterine displacement. The intraoperative period was uneventful, and the bilevel positive airway pressure was not required. Thereafter, she was admitted to the obstetric ward; for postoperative pain management, she was administered acetaminophen (1000 mg) every 6 h, ibuprofen (600 mg) every 6 h, and if needed, oxycodone (5 mg) every 6 h. She was provided with early in-hospital physical therapy management, with follow-up at home. After the patient regained mobility on postoperative day 1, she was referred to a physiotherapist who provided her with a set of exercises to perform at home. She was followed up for 6 months. At 3 months, the patient still had mild leg pain. After 6 months, the patient did not have any worsening of symptoms or relapsing episodes. She further reported walking without aid and resuming her normal activities. The patient reported being satisfied with the medical care received.

## 3. Discussion

In general anesthesia, the most important considerations are airway management, hyperkalemia, and muscle relaxation. Patients with TM may require the administration of succinylcholine since they are more sensitive to muscle relaxants than healthy patients. Further, patients who present for surgery with symptoms of acute TM may develop hemodynamic instability due to autonomic dysfunction [8]. Acute spinal shock leading to hypotension as well as hypertension and bradycardia are anticipated complications under general anesthesia. Moreover, there is a risk of aspiration resulting from gastroparesis during general anesthesia [7].

The inflammatory nature of TM and BD may lead to difficulties in intubation and ventilation due to ulceration of the oropharyngeal space [8]; intubation itself is a risk factor for ulceration in patients with BD. Thus, these neurological and dermatologic issues present a challenge during anesthesia [9]. Some of these skin manifestations include mucocutaneous disorders characterized by recurrent aphthous ulcers, ulcers in the external genitalia, and acneiform skin lesions.

In three of the previously reported cases, general anesthesia was performed. The first case was of a pregnant woman with preexisting TM who received general anesthesia for primary cesarean section. Upon presentation, the patient had no neurological signs or symptoms, and the surgery and postoperative period were unremarkable [6]. Another pregnant woman with BD was asymptomatic upon her presentation for labor. The perioperative period was unremarkable; intubation went smoothly, and the patient did not develop any neurological complications postoperatively [9]. Thus, none of these cases presented with severe neurological symptoms, and neuroaxial anesthesia was not preferred regardless of the symptoms.

In another report, a 32-year-old pregnant woman presented in the 32^nd^ week of gestation with sensory disturbance, hyperreflexia, and paraplegia due to TM. Recurrent rate decelerations were noted in the fetus, and she was transferred to the surgical room for an urgent cesarean section [10]. Neuroaxial anesthesia was considered as an option, but the presumed risks of neurological complications, coupled with the risks of aspiration, were very high. General anesthesia was administered via endotracheal intubation. However, the patient required supportive therapy for acute neurological complications due to her underlying condition [10]. These cases suggest that general anesthesia could be performed in pregnant women with BD or TM undergoing cesarean section. The neuroaxial approach is not excluded as an option; however, since the preferred approach is general anesthesia, it remains unreported.

Only one published case reported the use of spinal anesthesia during a pregnancy complicated by a history of BD. Before the surgery, a multidisciplinary team performed a comprehensive examination of the patient to evaluate the neurological symptoms and history; the results were unremarkable, as were the perioperative stage and postoperative recovery [11].

In this case, we highlighted a different approach for anesthetizing patients with BD and described the safety of the neuroaxial approach. This approach has been avoided owing to the presumed safety issues regarding disease progression or worsening of neurological symptoms. In this case, epidural approach was selected by the patient, and it helped avoiding the various complications associated with general anesthesia. Although our patient was not strictly adherent to her physical therapy exercises, her condition did improve.

Nonetheless, BD, NBD, and TM, all increase the risk of complications and adverse events during anesthesia. Although most pregnant women with TM or BD receive general anesthesia [6, 9, 10], spinal anesthesia has also been shown to be safe [11].

In conclusion, anesthesia is a clinical challenge in pregnant women with TM, BD, or NBD for various reasons. The lack of clinical and empirical information as well as standardized, evidence-based protocols implies that the anesthesia approach in pregnant women with TM, BD, or NBD should always be individualized. Multidisciplinary involvement could facilitate the safe administration of anesthesia in these patients. Unfortunately, there are no adequate studies on the neuroaxial outcomes in patients. Nevertheless, the development of standardized recommendations and protocols could greatly improve the quality and outcomes of anesthesia in patients with TM, BD, or NBD. Further, epidural blocks could also be successfully and safely administered to patients having BD with TM.

## References

[1] Mendes D., Correia M., Barbedo M., Vaio T., Mota M., Gonçalves O. (2009). Behçet’s disease—a contemporary review. *Journal of Autoimmunity*.

[2] Verity D. H., Marr J. E., Ohno S., Wallace G. R., Stanford M. R. (1999). Behçet’s disease, the Silk Road and HLA-B51: historical and geographical perspectives. *Tissue Antigens*.

[3] Sfikakis P. P., Markomichelakis N., Alpsoy E. (2007). Anti-TNF therapy in the management of Behcet’s disease—review and basis for recommendations. *Rheumatology*.

[4] Bitik B., Ucar M., Tezcan M. E. (2013). Transverse myelitis in Behçet’s disease: a series of four cases and review of the literature. *Clinical & Experimental Rheumatology*.

[5] Kalra S., Silman A., Akman-Demir G. (2014). Diagnosis and management of Neuro-Behçet’s disease: international consensus recommendations. *Journal of Neurology*.

[6] Galang K. A. P., Lim C. G. L. (2016). Transverse myelitis preexisting in pregnancy. *Philippine Journal of Obstetrics and Gynecology*.

[7] Fleisher L. A., Roizen M. F., Roizen J. (2017). *Essence of Anesthesia Practice*.

[8] Lukovic E., Mankowitz S. K. W., Mankowitz S. K. W. (2018). Transverse myelitis. *Consults in Obstetric Anesthesiology*.

[9] Koca E., Şayan H. (2017). Management of anesthesia in a pregnant with Behçet’s disease. *Kafkas Journal of Medical Sciences*.

[10] Hunter S., Katz D., Riley K., Anderson M. (2018). Anaesthesia for an emergent caesarean section in a patient with acute transverse myelitis. *Journal of Obstetric Anaesthesia and Critical Care*.

[11] Metodiev Y., Walker T. (2018). Anaesthetic considerations in a parturient with Behcet’s disease. *International Journal of Obstetric Anesthesia*.

